# Multi-Omics Integration Reveals Only Minor Long-Term Molecular and Functional Sequelae in Immune Cells of Individuals Recovered From COVID-19

**DOI:** 10.3389/fimmu.2022.838132

**Published:** 2022-04-06

**Authors:** Zhaoli Liu, Gizem Kilic, Wenchao Li, Ozlem Bulut, Manoj Kumar Gupta, Bowen Zhang, Cancan Qi, He Peng, Hsin-Chieh Tsay, Chai Fen Soon, Yonatan Ayalew Mekonnen, Anaísa Valido Ferreira, Caspar I. van der Made, Bram van Cranenbroek, Hans J. P. M. Koenen, Elles Simonetti, Dimitri Diavatopoulos, Marien I. de Jonge, Lisa Müller, Heiner Schaal, Philipp N. Ostermann, Markus Cornberg, Britta Eiz-Vesper, Frank van de Veerdonk, Reinout van Crevel, Leo A. B. Joosten, Jorge Domínguez-Andrés, Cheng-Jian Xu, Mihai G. Netea, Yang Li

**Affiliations:** ^1^ Centre for Individualised Infection Medicine (CiiM), a joint venture between the Helmholtz Centre for Infection Research (HZI) and Hannover Medical School (MHH), Hannover, Germany; ^2^ TWINCORE Centre for Experimental and Clinical Infection Research, a joint venture between the Helmholtz Centre for Infection Research (HZI) and the Hannover Medical School (MHH), Hannover, Germany; ^3^ Department of Internal Medicine and Radboud Center for Infectious Diseases, Radboud University Medical Center, Nijmegen, Netherlands; ^4^ Instituto de Ciências Biomédicas Abel Salazar (ICBAS), University of Porto, Porto, Portugal; ^5^ Department of Laboratory Medicine, Laboratory for Medical Immunology, Radboud University Medical Center, Nijmegen, Netherlands; ^6^ Laboratory of Pediatric Infectious Diseases, Radboud Centre for Infectious Diseases, Radboud University Medical Center, Nijmegen, Netherlands; ^7^ Institute of Virology, University Hospital Duesseldorf, Medical Faculty, Heinrich Heine University Duesseldorf, Dusseldorf, Germany; ^8^ Department of Gastroenterology, Hepatology and Endocrinology, Hannover Medical School, Hannover, Germany; ^9^ Institute of Transfusion Medicine and Transplant Engineering, Hannover Medical School, Hannover, Germany; ^10^ Department for Immunology and Metabolism, Life and Medical Sciences Institute (LIMES), University of Bonn, Bonn, Germany

**Keywords:** COVID-19, SARS-CoV-2, convalescence, multi-omics, single-cell sequencing, DNA methylation

## Abstract

The majority of COVID-19 patients experience mild to moderate disease course and recover within a few weeks. An increasing number of studies characterized the long-term changes in the specific anti-SARS-CoV-2 immune responses, but how COVID-19 shapes the innate and heterologous adaptive immune system after recovery is less well known. To comprehensively investigate the post-SARS-CoV-2 infection sequelae on the immune system, we performed a multi-omics study by integrating single-cell RNA-sequencing, single-cell ATAC-sequencing, genome-wide DNA methylation profiling, and functional validation experiments in 14 convalescent COVID-19 and 15 healthy individuals. We showed that immune responses generally recover without major sequelae after COVID-19. However, subtle differences persist at the transcriptomic level in monocytes, with downregulation of the interferon pathway, while DNA methylation also displays minor changes in convalescent COVID-19 individuals. However, these differences did not affect the cytokine production capacity of PBMCs upon different bacterial, viral, and fungal stimuli, although baseline release of IL-1Ra and IFN-γ was higher in convalescent individuals. In conclusion, we propose that despite minor differences in epigenetic and transcriptional programs, the immune system of convalescent COVID-19 patients largely recovers to the homeostatic level of healthy individuals.

## Introduction

Coronavirus disease 2019 (COVID-19), caused by the novel severe acute respiratory syndrome coronavirus 2 (SARS-CoV-2), has resulted in a considerable global morbidity and mortality (1). The majority of infected individuals experience mild to moderate symptoms and recover within several weeks after infection (2). Earlier studies have reported that lymphopenia and a higher level of T cell exhaustion serve as the hallmarks of severe infection (3, 4). The release of inflammatory cytokines, e.g., interleukin (IL)-6, IL-1β, tumor necrosis factor (TNF)-α, and IL-8, tend to rise during this viral infection, determining severe inflammatory complications (5, 6). Elevated neutrophil count with higher neutrophil-to-lymphocyte ratio (NLR) as well as reduced monocyte, basophil, and eosinophil counts were also reported in peripheral blood from COVID-19 patients, with NLR being used as an early warning marker for COVID-19 severity (7).

Changes at the transcriptomic level were also observed in immune cells isolated from COVID-19 patients, with the response to interferon(IFN)-α and inflammatory pathways being significantly upregulated (8). On the other hand, during severe COVID-19 infection, patients experienced reduced IFN-α and IFN-β production and activity, which leads to a high viral load and imbalanced inflammatory response (9). Moreover, lower expression of HLA-DR molecules was observed on monocytes and dendritic cells from COVID-19 infected patients, a sign of immune paralysis (10–12).

SARS-CoV-2 also hijacks the host epigenetic processes and may subsequently alter the transcriptome to evade the host immune defense. Recent studies reported hypermethylation in the gene regions related to IFN response and significant hypomethylation in the gene regions related to inflammation and cytokine production in severe COVID-19 cases, resulting in the shutoff of antiviral IFN response, uncontrolled inflammation, and cytokine storm (13, 14). One recent study found that the DNA methylation signatures from 44 5’-C-phosphate-G-3’(CpG) sites could predict COVID-19 disease severity (15).

While much has been done to understand the activation of immune responses during SARS-CoV-2 infection, much less is known about the long-term immunological sequelae of these processes. On the one hand, COVID-19 induces a robust adaptive immune memory response, which is characterized by an increased number of effector and memory T cells and antibody-producing plasma cells (16). Whether the immunological effects of COVID-19 extend beyond adaptive immune memory and also incorporate changes in innate and heterologous adaptive immunity is not known. Multi-omics integration can provide a deeper understanding of the immune system (17). We, therefore, investigated whether COVID-19 continues to affect the immune system’s functioning after recovery. To this end, we integrated multi-omics studies and functional assays to explore if SARS-CoV-2 infection induces any persistent changes at the transcriptome, epigenome, or chromosome accessibility level in convalescent COVID-19 individuals.

## Methods

### Study Subjects

The ethical approval for the study was obtained from CMO Arnhem-Nijmegen (NL32 357.091.10). All participants gave written consent for their participation in the study. In total, 14 convalescent COVID-19 patients and 15 sex-matched healthy donors (controls) from Radboudumc, the Netherlands, participated in this study. Convalescent patients were included in the study based on self-reported symptoms and subsequent confirmation of the presence of IgA, IgG, and IgM antibodies against the SARS-CoV-2 Spike protein in the circulation. All controls were at healthy status during sampling and did not experience COVID-19 based on antibody level (**Figure S1**). At the time of inclusion, patients had no reported COVID-19 symptoms or any other infections. Inclusions took place between April and June of 2020, during the first wave of the pandemic in the Netherlands. The demographic and clinical characteristics of the participants are provided in **Table 1**.

**Table 1:** General characteristics of the study population.

	Convalescence		Control	P value
	(n=14)		(n=15)	
**Age/years** (mean ± sd)	60 ± 11		50 ± 14	0.054
**Gender** (# M/# F)	7/7		7/8	1
**BMI** (kg/m2)	22 ± 2.53		22 ± 1.85	0.97
				
**Symptom**	Fever and headache	12/14		
(# of having symptom/# all individuals)	sleepy and tired	7/14		
	no taste	6/14		
	no smell	5/14		
	cough	4/14		
	pain	4/14		
	weight loss	2/14		
	nausea	1/14		
	no appetite	1/14		

### Isolation and Cryopreservation of Peripheral Blood Mononuclear Cells (PBMCs)

Whole blood was diluted with phosphate-buffered saline (PBS), and PBMC isolation was performed by density gradient centrifugation using Ficoll-Paque (GE Healthcare, IL, USA). The PBMC fraction was collected and washed three times with PBS. The cells were resuspended in RPMI 1640 Medium (Dutch modification) (Thermo Fisher Scientific, MA, USA) supplemented with 1 mM sodium pyruvate (Thermo Fisher Scientific), 2 mM GlutaMAX supplement (Thermo Fisher Scientific), and 5 µg/mL gentamicin (Centraform, Netherlands). For DNA methylation, single cell RNA sequencing (scRNA-seq), and single cell ATAC sequencing (scATAC-seq) analysis, and future stimulation assays, cells were cryopreserved in RPMI containing 40% bovine calf serum (Cytiva, MA, USA) and 15% dimethyl sulfoxide (VWR, PA, USA).

### Flow Cytometry From Whole Blood

After erythrocyte lysis in isotonic NH4CL buffer and washing twice with PBS, total leukocytes were obtained. White blood cell counts were determined by a cell counter (Coulter Ac-T Diff^®^ cell counter; Beckman Coulter, CA, USA) and used to calculate the absolute numbers of CD45+ leukocytes identified by flow cytometry as previously described in detail (18). Briefly, half a million of total leukocytes per staining panel were used to analyze surface markers with the Navios™ flow cytometer (Beckman Coulter). Cells were transferred to a V-bottom 96-well plate and washed twice with PBS containing 0.2% bovine serum albumin (BSA; Sigma-Aldrich, St. Louis, USA), stained for 20 minutes at room temperature in the dark with the staining panels of interest and washed twice with PBS containing 0.2% BSA. The conjugated antibodies specific for human cells are given in **Table S1**. The gating strategy used in flow cytometry analyses were provided in **Figure S2**. Data analysis was performed using the Kaluza 2.1^®^ software (Beckman Coulter).

### scRNA Sequencing

Frozen cells were rapidly thawed at 37 °C and transferred into 50 mL centrifuge tubes. Subsequently, cells were counted using an automated cell counter (Thermo Fisher Scientific). An equal number of cells (3,300 per individual) from 4 different individuals were pooled together and loaded into a 10X Chromium Controller to generate Gel Beads-in-emulsion (GEMs). scRNA-seq libraries were generated according to the manufacturer’s instructions (Chromium Next GEM Single Cell 3’ Reagent Kits v3.1 (Dual Index) User Guide, Rev A, CG000315 Rev A). In brief, all the loading cells were separated into nanoliter-scale droplets. Within each droplet, cDNA was generated *via* reverse transcription, adding a cell barcoding sequence and unique molecular identifier (UMI) to each cDNA molecule. Library quality per pool was examined using the Agilent Bioanalyzer High Sensitivity DNA kit. Sequencing was carried out on NovaSeq 6000, having a 50,000 reads depth per cell. DNA was isolated from PBMCs and then subjected to genotyping by Illumina Global Screening Array Beadchip to demultiplex the pooled samples.

### scRNA Sequencing Data Analysis

The Cellranger (v.4.0.0) utility of 10X genomics was used to process the scRNA-seq data. The standard protocol of the Cellranger pipeline consists of mapping sequenced reads and measuring gene expression (19). The human reference genome (GRCh38) was used for mapping sequence reads. Later, demultiplexing of individuals mixed in the same pool was done by using the genotype-free clustering method souporcell (20). Further downstream analyses were performed using the Seurat package V4 (21) in R following its default pipelines, i.e., quality filtering, data normalization, and scaling. During quality filtering, mitochondrial and ribosomal genes along with cells having mitochondrial reads > 25% and expressed genes > 5000 or < 250 were removed. Data normalization was done using a global-scaling normalization method, namely “LogNormalize”. Subsequently, data was scaled prior to dimensionality reduction through principal component analysis (PCA) using the top 2,000 variable features detected using the vst method. Uniform Manifold Approximation and Projection (UMAP) technique was utilized for cell clustering. The “FindAllMarkers” function was used for identifying marker genes for each cluster. Using publicly available information, each cluster was annotated based on top marker genes (having the lowest adjusted p-value and positive logFC). Differentially expressed genes (DEGs) between convalescent COVID-19 patients and controls were detected through the “FindMarkers” function using the MAST test (22) adjusted by age and sex. Genes that were expressed in at least 10% cells and with adjusted P-value (after Bonferroni *post-hoc* correction) < 0.05 were considered significant. Gene Ontology (GO) enrichment analysis of significant DEGs was done using the “clusterProfiler” package in R (23). Later, for cross-validation, a matrix containing the total gene count per sample was generated from Seurat by summing up all the read counts per gene, and the pseudo-bulk RNA analysis was performed through DESeq2 (24).

### scATAC Sequencing

Cryopreserved PBMCs were thawed at 37°C and transferred into 50 mL centrifuge tubes. PBMCs were washed in PBS through centrifugation at 400G for 5 minutes at 4 °C and lysed for 3 minutes on ice. After discarding the supernatant, lysed cells were diluted within 1× diluted nuclei buffer (10X Genomics) prior to counting with trypan blue using a Countess II FL Automated Cell Counter to validate lysis. An equal number of nuclei (approximately 3000 nuclei per sample) from four individuals were pooled together and then loaded into the Chromium Next GEM Chip H based on the user guides from 10X genomics (Chromium Next GEM Single Cell ATAC Reagent Kits v1.1 User Guide, CG000209 Rev D). After breaking the emulsion, the barcoded tagmented DNA was purified and amplified for sample indexing and generation of scATAC-seq libraries. The final libraries were quantified using the Agilent Bioanalyzer High Sensitivity DNA kit. Sequencing was performed on NovaSeq 6000 with a depth of 25,000 reads per nuclei.

### scATAC Sequencing Data Analysis

Using the cellranger-atac mkfastq (10X Genomics, v.1.2.0) utility, raw sequencing data were converted to FASTQ format. Reads were aligned based on the human reference genome (GRCh38). Demultiplexing and doublets removal was done using souporcell (20). Each scATAC-seq library fragment file was utilized further for downstream analysis. Employing the ArchR package (v.1.0.1) within R, quality control (cells having a transcription start site (TSS) enrichment score < 4 and unique nuclear fragments < 1000 were filtered), dimensionality reduction (iterativeLSI function, default parameters), harmony batch correction (25) (addHarmony function, default parameters), clustering (addClustersfunction, method = “Seurat”, resolution = 1, UMAP) and cluster visualization (addUMAP function) were done (26). The default parameter of ArchR was used to calculate gene expression by computing a “gene score’’ based on chromatin accessibility within a gene body and distally & proximally from the transcription start site (TSS). A gene score matrix was created using the gene score profiles for all cells. Later, the wilcoxon rank-sum test was used to identify cell type specific marker genes. Gene having adjusted *P-value* (Benjamini-Hochberg) < 0.05 and logFC >= 0.05 were considered as marker genes. Each cluster was annotated based on cell type specific marker genes and cluster specific marker genes retrieved from scRNA-seq data. Further, using the method (“addGeneIntegrationMatrix” function) developed by Stuart (27), the gene score matrix was integrated with scRNA expression data. MACS2 (28) strategy (getMarkerFeatures function, useMatrix = “PeakMatrix”) was adapted on an integrated dataset to call peaks in each cluster. Peaks having logFC greater than 0.05 and *P-value* < 0.05 were considered as marker peaks. For cross-validation, a peak matrix per sample was generated, and the pseudo-bulk ATACseq analysis was performed through ArchR.

### *In Vitro* Measurement of Interferon Response

Six healthy controls and six convalescent COVID-19 patients were used to assess interferon response. Cryopreserved PBMCs were stimulated with 10 ng/ml recombinant human IFN-α2 (R&D Systems), 1000 U/ml IFN-β (R&D Systems), and 50 ng/ml IFN-γ1b (Miltenyi Biotec, Germany) for 24 hours. Stimulations were performed in U-bottom 96-well tissue culture plates with 5x10^5^ PBMCs per well. RNA isolation was carried out using the RNeasy Mini kit (Qiagen, Germany), and cDNAs were generated with the iScript cDNA synthesis kit (Bio-Rad Laboratories, CA, USA) according to the instructions of the manufacturers. RT-qPCR for the interferon-response genes *IFI44L, IFI6, IRF3, IRF7, IRF9, ISG15*, and *OAS2* was performed with StepOnePlus PCR System (Applied Biosystems, MA, USA). HPRT1 was used as the housekeeping control gene. Primers and reaction conditions are provided in **Tables S2, S3**.

### DNA Methylation Analysis

DNA was isolated from whole blood using QIAamp DNA Micro Kit (Cat: 56304, Qiagen, Hilden, Germany), and the concentration was determined using a NanoDrop spectrophotometer at 260 nm. The high-quality DNA from 20 samples (11 convalescent COVID-19 and 9 controls) were obtained successfully for the genome-wide DNA methylation profiling through the Illumina Infinium^©^ MethylationEPIC array (~850,000 CpG sites). The DNA methylation values were gained from the raw IDAT files using the minfi package in R (v.4.0.3) (29). Initially pre-processing was performed to filter bad quality probes with a detection *P-value* > 0.01, cross-reactive probes, polymorphic probes (30), and probes in the sex chromosome. Subsequently, after stratified quantile normalization (31), the comparison was made between convalescent COVID-19 individuals and controls to detect the relative proportion of cell types and differentially methylated CpG sites. Based on methylation value, cell proportion was estimated using modified Housman’s method implemented in the estimateCellCounts2 function of the FlowSorted.Blood.EPIC R package (32). Later, differential analysis of cell-type proportions was done using the regression model of the DirichletReg package of R. Differentially methylated CpG sites were detected by a linear regression model using the limma R package (33), with age and sex as covariates. The Spearman correlation was calculated between the cell proportion and the top 150 CpG differentially methylated sites. DMRs analysis was done by using combp R package (34).

### PBMC Stimulations and Cytokine Measurements

5x10^5^ PBMCs per well were seeded in U-bottom 96-well tissue culture plates (Greiner Bio-One, Austria) and stimulated with 10 ng/ml lipopolysaccharides (LPS) derived from Escherichia coli O55:B5 (Sigma-Aldrich, MO, USA), 10^6^/ml heat-killed Staphylococcus aureus, 10^6^/ml heat-killed Candida albicans, R848 (*InvivoGen*), or heat-inactivated(30 minutes, 60°C) SARS-CoV-2 Wuhan-Hu-1 strain (NRW-42 isolate, GISAID accession number: EPI_ISL_425126) (35) with 1 µg/ml soluble purified mouse anti-human CD28 (BD Biosciences, New Jersey, USA), for 24 hours and 7 days in the presence of 10% pooled human serum. A control condition without any stimulant was included. Cells were incubated at 37°C with 5% CO2. Stimulations with R848 and SARS-CoV-2 were performed later with cryopreserved cells, while the rest of the stimulations were done with freshly isolated PBMCs. Concentrations of TNF-α, IL-6, IL-1β, IL-1Ra, and IFN-γ in culture supernatants were determined using DuoSet ELISA kits (R&D Systems, MN, USA). IFNα concentrations were determined using VeriKine Human Interferon Alpha ELISA Kit (PBL Assay Science, NJ, USA) according to the manufacturer’s instructions. Supernatants from 7 days stimulations were used for IFN-γ, and the remaining cytokines were measured in 24-hour supernatants.

## Results

### Study Design

To characterize the immunological features of convalescent COVID-19 patients, we collected blood from 14 convalescent COVID-19 patients and 15 healthy donors. At the time of sampling, none of the control individuals experienced any symptoms or signs of infection, including COVID-19. All convalescent COVID-19 patients had recovered from SARS-CoV-2 infection. Out of the 14 patients, only one convalescent COVID-19 patient had experienced a severe COVID-19 and was admitted to an intensive care unit (ICU), while the remaining 13 experienced mild infections. The recovery time for most of the convalescent COVID-19 patients (9 out of 14) was ca. one month, only one patient with one-week recovery time and two patients with around two-month recovery time. Sex was distributed almost equally in both convalescent and healthy individuals. The mean age of convalescent COVID-19 patients and control individuals were 60 ± 11 years and 50 ± 14 years, respectively (**Table 1**).

**Figure 1:**
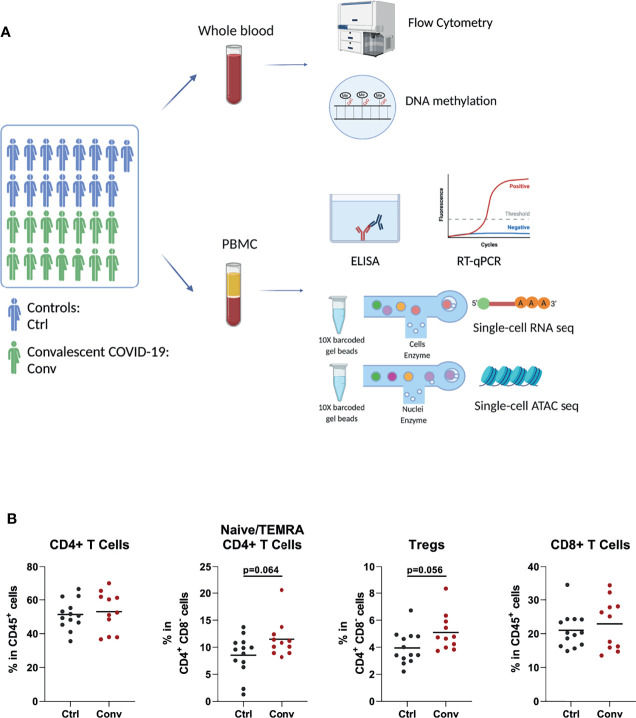
Study design and immune cell populations of healthy controls and convalescent COVID-19 patients. **(A)** The summary of the experiments performed in the study. Blood was drawn from 15 healthy controls and 14 convalescent COVID-19 patients. Flow cytometry, RT-qPCR, and ELISA were performed to determine the cell counts, assess interferon stimulated gene expressions, and measure the cytokine levels in culture supernatants, respectively. Single-cell RNA sequencing was used to determine the transcriptome. Single-cell ATAC sequencing and whole-genome methylation assay were performed to analyze the epigenetic differences between convalescent COVID-19 individuals and controls (This figure was created with BioRender.com). **(B)** Dot plots showing proportions of T cell subsets between the convalescent COVID-19 individuals and controls from flow cytometry. Mann-Whitney test was used to analyze the differences between controls (black dots) and convalescent patients (red dots).

To explore the effect of the SARS-CoV-2 infection on the immune system after recovery, we have performed a comprehensive set of assays and analyses, which were summarized in **Figure 1A**.

### Subtle Differences of Immune Cell Composition in COVID-19 Convalescence

Since acute SARS-CoV-2 infection leads to striking changes in immune cell types in the blood, flow cytometry analysis was performed to assess whether the abundance of various immune cell subsets in convalescent COVID-19 individuals is still different from those of healthy controls. The absolute numbers and percentages of most immune cell subsets, such as neutrophils, natural killer (NK) cells, B cells, and T cells, were similar between convalescent patients and controls (**Figures S3A–C**). Although COVID-19 leads to a dramatic decline in CD4+ T and CD8+ T cells in the acute phase, these cell populations in convalescent patients were not significantly different from those of uninfected people, indicating the full recovery from lymphopenia (**Figure 1B**). We observed that regulatory T cells (Treg) and CD4+ naive/terminally differentiated effector memory cell (naïve/TEMRA) populations were somewhat more abundant in the convalescent COVID-19 individuals compared to controls, but these differences were not statistically significant (**Figure 1B**).

### Single-Cell RNA-Seq and ATAC-Seq Analysis of PBMCs in Convalescent COVID-19 Individuals

**Figure 2:**
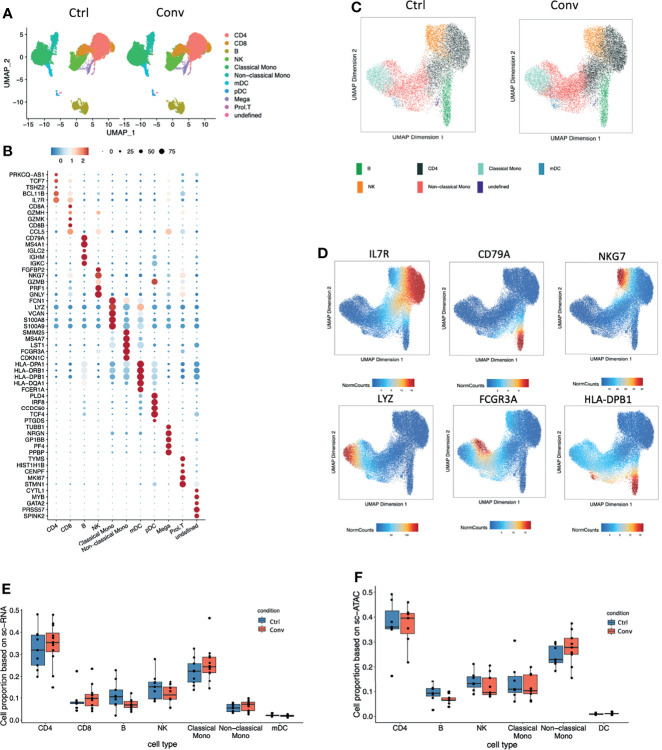
Single-cell transcriptome and epigenome analysis defined major immune cell subsets in convalescent COVID-19 individuals. **(A)** A total of 41486 and 25266 single-cell transcriptomes from COVID-19 convalescent and control samples were obtained respectively. Using the uniform manifold approximation and projection (UMAP) method, we captured 11 major cell types based on the canonical markers in the dot plots (B). **(B)** Dot plot showing the expression level of the marker genes in each cell type. Dot size reflects the proportion of cells expressing the indicated gene; the color encodes the average expression level (low=blue, high=red). **(C)** A total of 21102 and 17084 nuclei from 9 COVID-19 convalescent and 7 control samples were obtained respectively. Seven major cell types were captured based on the canonical markers shown in the feature plot(D). **(D)** The UMAP showing the labelled gene score for each cell. Blue represents the minimum gene score while red represents the maximum gene score for the given gene. The minimum and maximum scores are shown in the bottom of each panel. The gene of interest are shown in the upper of each panel. Cell proportions from **(E)** single-cell RNA sequencing and **(F)** single-cell ATAC dataset between convalescent COVID-19 and controls in the seven main cell types, CD4+ T cells, CD8+ T cells, B cells, NK cells, classical monocytes, non-classical monocytes, and mDCs.

Next, we performed an integrative analysis of scRNA-seq and scATAC-seq in convalescent and healthy individuals. For scRNA-seq analysis, we examined 66,753 single cells from 21 individuals (12 convalescent and 9 controls) after QC (**Figure S4A**). Uniform manifold approximation and projection (UMAP) was used for cell clustering. In total, we detected 11 immune cell types, namely, CD4+ T cells (IL7R+), CD8+ T cells (CD8A+CD8B+GZMK+), B cells (CD79A+), NK cells (NKG7+GZMB+), classical monocytes (CD14+LYZ+), non-classical monocytes (FCGR3A+), platelets (PPBP+), and monocyte-derived dendritic cells (mDCs, HLA-DP^high^, HLA-DR^high^) (**Figures 2A, B**). Subsequently, we analysed 38,186 nuclei from 16 individuals (9 convalescent and 7 controls) in scATAC-seq analysis (**Figures 2C, D** and **Figure S4B–D**). It is pertinent to note that a strong correlation of the marker genes between scRNA-seq and scATAC-seq analysis was detected (**Figure S4E**). Subsequently, we compared the cell compositions of both scRNA-seq and scATAC-seq. No statistically significant difference was detected between the convalescent COVID-19 patients and controls (**Figures 2E, F**). This supports the findings of the flow cytometry measurements showing full recovery of the immune cell subsets.

### Pathways of Antigen Processing and Presentation *via* MHC II Remain Downregulated in Convalescent COVID-19 Individuals at Transcriptional Level

To identify if any differences exist at the transcriptomic level between immune cells of convalescent COVID-19 patients and controls, we performed a differential expression analysis. After correcting for age and sex, 907 differentially expressed genes (DEGs) were identified with false discovery rate (FDR) < 0.05 and absolute log-fold change (logFC) > 0.05 (**Figure S5A**). DEGs were found predominantly in monocytes (both classical (DEGs=579) and non-classical (DEGs=62)) and CD4+ T (DEGs=172) cells. Moreover, we assessed the number of DEGs at different logFC thresholds: we identified 293 DEGs with FDR < 0.05 and absolute logFC > 0.1, and 30 DEGs with FDR < 0.05 and absolute logFC > 0.25. Although the number of DEGs decreased with higher logFC thresholds, most of them predominantly existed in monocytes and CD4+ T cells (**Figure S5A**). This indicates that the observed differences in the transcriptional profile of convalescent COVID-19 patients were mainly in monocytes and CD4+ T cells. Additionally, we compared the peak accessibility in each cell type based on scATAC-seq data. After multiple testing correction, none of the peaks were significant, which indicated that the open chromatin accessibility between convalescence patients and healthy controls did not show significant difference. The differential expressed genes were mainly found in classical monocytes. In classical monocytes, for peaks in the proximity (500kb) of up-regulated DEGs in convalescence, 7205 peaks were up-regulated (mean log2FC = 1.077) in convalescence while 7371 peaks were down-regulated (mean log2FC = -1.073). For peaks in the proximity (500kb) of down-regulated DEGs in convalescence, 10252 peaks were down-regulated (mean log2FC = -1.078) in convalescence while 9777 peaks were up-regulated (mean log2FC = 1.086). None of these peaks were significantly different between convalescence and controls (P-value _FDR adjusted_ > 0.5).

**Figure 3:**
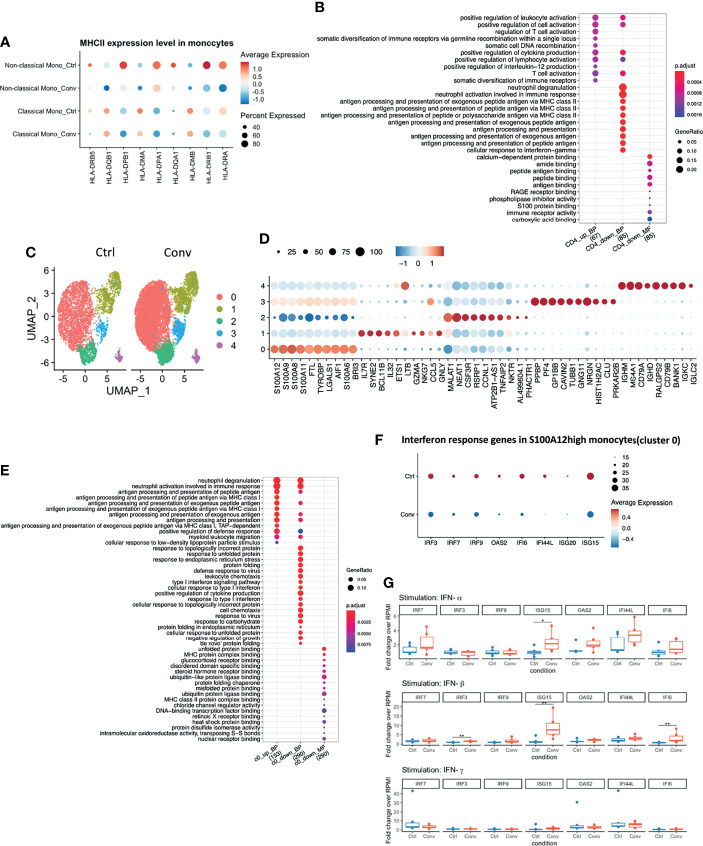
Antigen processing and presentation *via* MHC II were downregulated in convalescent COVID-19 individuals, while baseline expressions of Interferon-Stimulated Genes (ISGs) in PBMCs are comparable to uninfected individuals. **(A)** Dot plot showing the MHC II expression levels across different conditions. The size of the dot depicts the percentage of cells within classical monocytes or non-classical monocytes; the color encodes the average expression level (low=blue, high=red). **(B)** Dot plot showing the GO enrichment analysis of DEGs in CD4+ T cells. The color and size of the dot indicate the significance (adjusted *P-value*) and the percentage of DEGs in the given GO term, respectively (BP, biological process, MF, molecular function). Top 10 enriched categories were shown. **(C)** UMAP of single-cell RNA sequencing data in classical monocytes in convalescent COVID-19 (n = 11) and control (n=9) samples. 5 cell clusters were identified. **(D)** Dot plot showing the top 10 markers for each cell cluster using the Wilcoxon Rank Sum test. The 0-4 in the y-axis means the cell clusters from(C). The color and size of the dot indicate the gene expression level (low=blue, high=red) and percentage of cells which expressing these markers. **(E)** Dot plot showing the GO enrichment categories of the DEGs found in each cluster. **(F)** Dot plot showing the interferon response genes expression levels across different conditions. The size of the dot depicts the percentage of cells expressing the indicated genes within cluster0; the color encodes the average expression level (low=blue, high=red). **(G)** Box plots showing the gene expressions of the seven interferon-stimulated genes (*IRF7, IRF9, IRF3, ISG15, OAS2, IFI44L*, and *IFI6*) 24 hours after stimulation with recombinant human IFN-α, IFN-β, and IFN-γ. Fold changes over RPMI (unstimulated condition) were reported. Mann-Whitney test was used to analyze the differences between controls and convalescent patients (n = 6, *P value < 0.05, **P value < 0.001).

Differential expression analysis in the scRNA-seq revealed that MHC class II genes, such as *HLA-DRB5, HLA-DPB1, HLA-DPA1, HLA-DRB1*, and *HLA-DRA*, were downregulated in convalescent COVID-19 individuals (**Figure 3A**). Moreover, antigen processing and presentation *via* the MHC II pathways were downregulated in CD4+ T cells (**Figure 3B**). On the other hand, no significant difference in the surface expression of HLA-DR in CD4+ T cells and monocytes between healthy and convalescent individuals was observed (**Figure S5B**).

### Interferon Pathway in Convalescent COVID-19 Patients and Healthy Volunteers

Since we identified DEGs mainly in classical monocytes (**Figure S5A**), we performed a sub-clustering analysis of these cells. Initially, using UMAP, we captured 6 sub-clusters in classical monocytes. Further inspection revealed that one healthy control, HC04, was markedly different from others (**Figures S5C, D**). Classical monocytes-specific pseudo-bulk RNA analysis also showed that HC04 was separated from the main group in a PCA plot (**Figure S5E**). Therefore, we temporarily removed HC04 while performing differential expression analysis within the classical monocytes. After discarding HC04, we re-performed sub-clustering and detected five sub-clusters (**Figure 3C**). The top markers for each sub-cluster are shown in **Figure 3D**. DEGs between convalescent individuals and controls mainly occurred in cluster 0 (S100A^high^) and cluster 1 (IL7R^high^) with FDR < 0.05 and logFC > 0.05 (**Figure S5F**). Subsequently, GO enrichment analysis was performed using the DEGs present in cluster 0 (812 DEGs) and cluster 1 (33 DEGs), respectively. The upregulated DEGs in convalescent individuals were enriched in antigen processing and presentation *via* MHC I. while the down-regulated DEGs were enriched in response to the interferon pathway in the S100A^high^ classical monocytes. No enriched pathway was found in DEGs from cluster 1 (**Figure 3E**). The genes engaged in the down-regulated interferon response pathway included interferon regulatory factor 3 (*IRF3*), *IRF7*, *IRF9*, 2’-5’-Oligoadenylate Synthetase 2 (*OAS2*), interferon-alpha inducible 6 (*IFI6*), interferon-alpha inducible ligand 44 (*IFI44L*), interferon-stimulated gene 20 (*ISG20*), and *ISG15* (**Figure 3F**). These genes were thus selected for the *in vitro* validation of the sequencing results.

IFN production constitutes the major first line of defense against viruses. Type I and type II IFNs induce hundreds of antiviral effectors, or ISGs, to achieve a cell-intrinsic state of viral resistance (36). Since IFN response pathway genes were downregulated in monocytes of convalescent patients, we hypothesized that expressions of those genes would be different between healthy and convalescent COVID-19 individuals after stimulation with IFN-α, IFN-β, and IFN-γ. Therefore, PBMCs of 6 healthy and 6 convalescent individuals were stimulated with type I and II interferons, and genes involved in IFN response were analyzed *via* RT-qPCR. The baseline gene expressions were not different between controls and convalescent individuals (**Figure S5G**). On the other hand, we found that expression of three ISGs, namely, *IRF3*, *ISG15*, and *IFI6*, were significantly higher in convalescent COVID-19 individuals after stimulation with IFN-β, IFN-α/β, and IFN-β, respectively (**Figure 3G**), indicating that the degree of induction by type I IFNs is higher for some of the IFN regulated genes in convalescent individuals compared to healthy volunteers. We performed pseudo-bulk RNA analysis between convalescent COVID-19 individuals and controls and found no significant difference, in line with the RT-qPCR results from non-stimulated PBMCs (**Table 2**). Consistently, pseudo-bulk ATAC analysis also revealed no significant difference in the chromatin accessibility of ISGs associated with the interferon response pathway in PBMC between conditions (**Table 2**). These results suggest that the down-regulation of the interferon pathway is specific to monocytes, while at the PBMC level, this pathway is not changed.

**Table 2:** Expressions of interferon-stimulated genes analyzed by pseudo-bulk RNAseq and pseudo-bulk ATACseq.

Gene	pseudo bulk RNA		pseudo bulk ATAC	
	log2FC	raw P	log2FC	raw P
*ISG15*	0.66	0.055	0.14	0.513
*IFI6*	1.04	0.021	0.04	0.693
*IFI44L*	1.18	0.285	0.31	0.048
*IRF7*	0.41	0.262	-0.29	0.249
*OAS2*	0.46	0.314	-0.46	0.088
*IRF9*	0.16	0.617	0.05	0.903
*IRF3*	-0.006	0.984	0.17	0.209

*Wald test was used to analyze the differences between controls and convalescent patients.

### The Differences in DNA Methylation Sites in Convalescent COVID-19 Patients Mainly Originated From Monocytes and CD4+ T Cells

**Figure 4:**
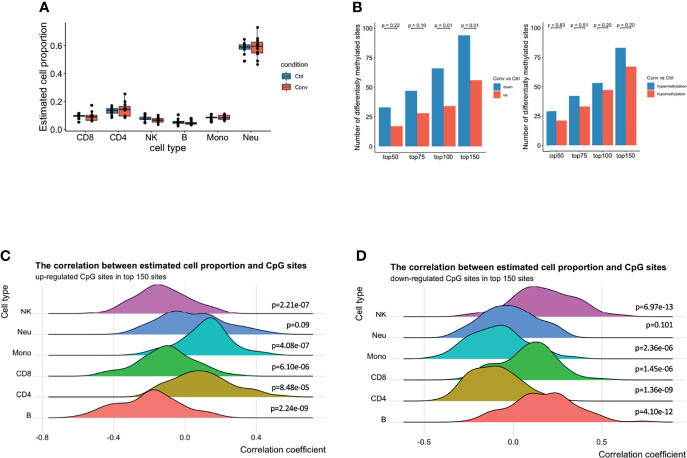
A predominance of down-regulated methylation sites was observed in convalescent COVID-19 patients, mainly originating from monocytes and CD4+ T cells. **(A)** Box plot showing the estimated cell proportions in convalescent COVID-19 (n = 11) and control (n = 9) samples based on the methylation. No significant difference was observed in estimated cell proportions between the two conditions. **(B)** The number of up-/down-regulated and hyper/hypomethylated CpG sites in top 150 differentials methylated CpG sites ordered by raw *P value* from the linear regression model. A preponderance of hypermethylated sites and down-regulated methylation value was consistently observed at various thresholds compared with those in healthy controls. Fisher’s exact test was used for the statistical analysis. **(C)** The density ridgeline plots showing the correction between the estimated cell proportions and the up-regulated CpG sites in the top 150 differentially methylated CpG sites. **(D)** The density ridgeline plots showing the correction between the estimated cell proportions and the down-regulated CpG sites in the top 150 differentially methylated CpG sites. One side Wilcoxon test was used to calculate the significance of the correlation.

Earlier studies have reported that the host cell epigenetic landscape of DNA methylation changes during SARS-COV2 infection (14). Therefore, we performed DNA methylome profiling from high-quality DNA of 20 whole blood samples (11 convalescent COVID-19 and 9 controls). Initially, pre-processing of the dataset scanned 794,745 good-quality probes. Cellular deconvolution analysis using modified Housman’s method (32, 37, 38) detected six cell-types, namely NK, CD8+ T, CD4+ T, B cells, neutrophils, and monocytes, which were present in both convalescent COVID-19 individuals and controls. The relative proportion of these six cell types did not significantly differ (**Figure 4A**). This result, consistent with our flow cytometry, scRNA-seq and scATAC-seq analyses, indicated that the cell proportions returned to normal levels after recovery from SARS-CoV-2 infection.

Furthermore, epigenome-wide association analysis (EWAS) was performed to assess the difference in genome-wide DNA methylation profiles between convalescent COVID-19 individuals and controls. No significant differentially methylated CpG sites (DMSs) (FDR < 0.05) were identified (**Figures S6A-C**). Subsequently, to detect any minor changes at the molecular level, we scanned the top 50, 75, 100, and 150 DMSs. A prevalence of hypermethylated and demethylated CpG sites was consistently observed at these top DMSs in convalescent COVID-19 patients compared with those in healthy controls (**Figure 4B**). Thus, genome-wide DNA methylation profiles of convalescent COVID-19 individuals show only minor differences compared to controls.

Next, we aimed to detect individual cell types where the methylation signal was the strongest. We performed a correlation analysis between the top 150 DMSs (94 downregulated and 56 upregulated) having the lowest *P-value* and the estimated cell proportions from the above-mentioned deconvolution analysis. We observed that the upregulated CpG sites were positively correlated with CD4+ T cells, monocytes, and neutrophils (**Figure 4C**). Conversely, the downregulated CpG sites showed a negative correlation with CD4+ T cells, monocytes, and neutrophils (**Figure 4D**), suggesting that majority of the top differentially DNA methylation signatures in convalescent COVID-19 individuals mainly originated from monocytes and CD4+ T cells, and somewhat less neutrophils. These results could be replicated using the top 100 or top 75 DMSs (**Figures S6D, E**). However, we could not identify enriched epigenetic changes around the DEGs (+/- 250kb window) in monocytes observed from the scRNA-seq, compared to randomly selected CpG sites (**Figure S6F**).

Differential methylation regions (DMRs) have identified 30 significant DMRs between convalescent COVID-19 individuals and controls (**Table S4**). The genes annotated to these DMRs were enriched in the following biological pathways: glycosphingolipid biosynthesis, telomere maintenance, recognition of DNA damage, sialic acid metabolism, chromosome maintenance and TGF-Ncore.

### Cytokine Production Capacity Is Not Impaired in People Recovered From COVID-19

**Figure 5:**
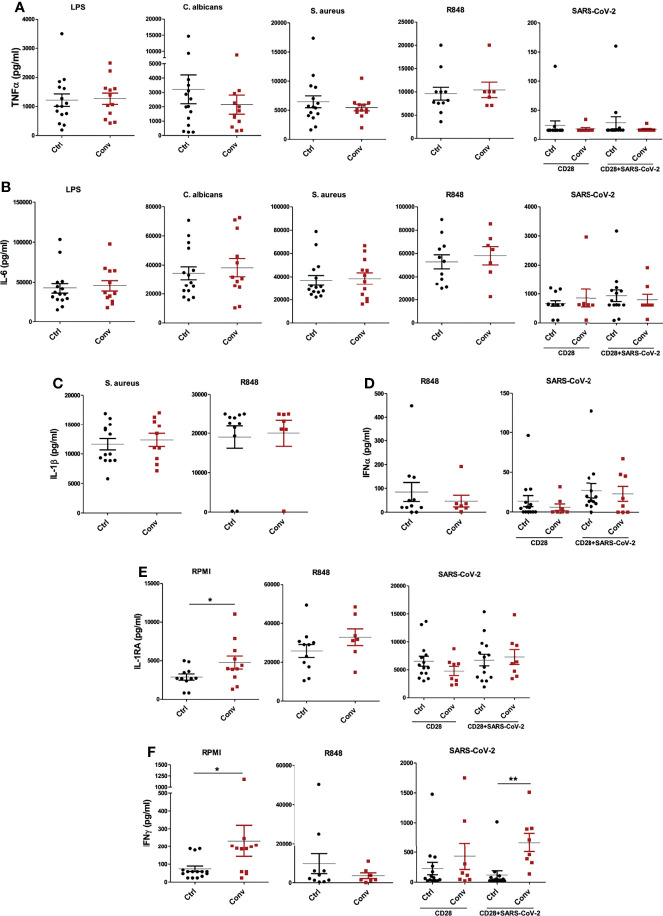
Cytokine production capacity is intact in people recovered from COVID-19. The concentrations of **(A)** TNF-α and **(B)** IL-6 after stimulation of PBMCs with LPS, *C. albicans*, S. aureus, R848, and heat-inactivated SARS-CoV-2. **(C)** IL-1β production following S. aureus and R848 stimulation. **(D)** IFNα levels after incubation with R848 and heat-inactivated SARS-CoV-2. **(E)** IL-1Ra and **(F)** IFN-γ at the baseline (RPMI condition) and stimulation with R848 and SARS-CoV-2. TNF-α, IL-6, IL-1β, IFNα and IL-1Ra productions were measured after 24 hours, while IFN-γ was measured after 7 days of incubation. Stimulations with R848 and SARS-CoV-2 were performed later with cryopreserved cells, while the rest of the stimulations were done with freshly isolated PBMCs. Mann-Whitney test was used to analyze the differences between controls and convalescent patients (*P value < 0.05, **P value < 0.001).

Lastly, we measured the cytokine production capacity of PBMCs upon incubation with different viral, bacterial, and fungal stimuli to assess whether the differences in transcriptome influence immune responses upon recovery from COVID-19. PBMCs were either left unstimulated or stimulated with LPS, *S. aureus*, *C. albicans*, viral RNA mimic R848, and heat-inactivated SARS-CoV-2 (original Wuhan-Hu1 strain) for 24 hours to measure TNF-α, IL-6, IL-1β, IFNα and IL-1Ra and seven days to measure IFN-γ. Anti-CD28 agonistic antibodies were combined with inactivated SARS-CoV-2 to better activate T cell responses, particularly in convalescent COVID-19 patients. TNF-α and IL-6 production upon different stimuli were similar between healthy individuals and convalescent COVID-19 patients (**Figures 5A, B**). Another proinflammatory cytokine, IL-1β, was also produced similarly in healthy and convalescent individuals upon R848 and *S. aureus* stimulation (**Figure 5C**). Furthermore, R848 and SARS-CoV-2 induced IFNα production from PBMCs did not differ between the groups (**Figure 5D**). Intriguingly, baseline IL-1Ra secretion of convalescent COVID-19 patients was significantly higher than healthy controls, although IL-1Ra production following R848 or heat-inactivated SARS-CoV-2 stimulation was similar (**Figure 5E**). Furthermore, PBMCs from convalescent individuals produced significantly more IFN-γ without any stimulation (**Figure 5F**). As expected, incubation of convalescent COVID-19 patient PBMCs with heat-inactivated SARS-CoV-2 resulted in higher IFN-γ production, indicating the presence of T cell memory after recovery. Lastly, IFN-γ secretion did not differ between healthy and convalescent upon incubation with viral RNA mimic R848 (**Figure 5F**).

## Discussion

In the present study we provided a detailed insight into the immunological and molecular features of immune responses in the convalescent COVID-19 individuals by integrating multi-omics data. Furthermore, we assessed whether SARS-CoV-2 infection influences immune functions in terms of *in vitro* cytokine production capacity of recovered individuals. We found only minor differences in the transcriptome and DNA methylation profiles of immune cells in convalescent COVID-19 individuals, and these differences were consistently more pronounced in monocytes and CD4+ T cells compared to other immune cell populations. However, these changes did not seem to affect the cytokine production capacity of immune cells, although they were accompanied by a higher homeostatic release of IL-1Ra and IFN-γ.

Results obtained from flow cytometry, scRNA-seq, scATAC-seq, and DNA methylation analyses consistently revealed that immune cell proportions were at the normal levels in convalescent COVID-19 individuals within one-week to 6-month timeframe. One earlier study reported a higher percentage of CD4+ T cells and DCs and lower numbers of NKT-like cells in patients two months after COVID-19 recovery compared to uninfected controls, while other cell types remained similar between groups (39). We observed no major difference in cell populations, only slightly higher levels of regulatory T cells (Tregs) in convalescent individuals. Tregs play a critical role in the maintenance of self-tolerance and immunological homeostasis by negatively regulating the activation, proliferation, and effector functions of a wide variety of immune cells (6). They can also prevent cytokine storm (40) and repress the formation of lung inflammatory disorders (41, 42). Although there are conflicting reports, most studies report decreased circulating Treg levels, especially in severe COVID-19 cases (42). Thus, the comparable numbers of Tregs as well as other immune cell subsets between healthy and recovered COVID-19 individuals in our study suggest that the immune cells go back to the level of healthy individuals after recovery. We also found that naive/TEMRA cell subset was somewhat more abundant in convalescent patients, although not statistically significant. TEMRA cells are known to expand following pathogens, such as the Dengue virus, and play a protective role during infections (43). Increased ratio of CD4+ TEMRA cells in moderate and severe COVID-19 was previously reported (44). The same study also showed that CD4+ TEMRA population stayed higher even after patients’ recovery, however, sampling time after recovery was not indicated.

Transcriptomic analysis revealed that most of the DEGs were detected in classical monocytes. Although the differences between convalescent individuals and controls were modest, and we did not find a distinct expression in the surface HLA-DR expression of monocytes and CD4+ T cells, the downregulation of the HLA class II pathway in convalescent COVID-19 patients is important to be noted. Broad MHC II downregulation during COVID-19 infection was also reported in previous studies (45). Our findings suggest that the attenuated immune function *via* MHC II downregulation during viral infection is still present at the transcriptomic level after recovery, and that may influence the capacity of myeloid cells to respond during infections a long time after SARS-CoV-2 was eliminated. Moreover, sub-clustering revealed upregulated MHC class I-dependent antigen processing and presentation pathways and downregulated interferon pathway in classical monocytes. However, the baseline expression of interferon pathway genes was not significantly different in the entire PBMC population, as evident from RT-qPCR, pseudo-bulk RNA, and pseudo-bulk ATAC sequencing analyses. Thus, these two pathways seem to be differentially regulated only in monocytes of convalescent COVID-19 individuals and neither represent the changes in the PBMC population nor translate into impaired *in vitro* response to IFNs. Notably, type I IFNs led to a significant increase in *IRF3*, *ISG15*, and *IFI6* expressions in convalescent COVID-19 patients compared to healthy controls, suggesting a higher degree of activation. On the other hand, stimulation with different antigens did not result in significantly different cytokine production, showing that changes in interferon pathway related-gene activation do not influence cytokine production capacity, including IFNα, in recovered COVID-19 individuals.

A recent study reported important epigenetic and functional changes in monocytes isolated from convalescent COVID-19 patients, especially in the IL-1 pathway and chemokines, suggesting a trained immunity phenotype (16). We did not identify similar changes in our individuals, suggesting potential heterogeneity between different populations, disease severity, or sampling timepoints. Similarly, we did not find significant changes in CD8+ T cell transcriptome in convalescent COVID-19 individuals compared to healthy controls, although such changes were reported during disease course, including upregulated CD8 expression, and an hyperactivated and exhausted phenotype (46, 47). However, since CD8+ T cells are a major source of IFN-γ production (48), higher IFN-γ production of non-stimulated PBMCs in convalescent COVID-19 patients might indicate that this hyperactive state of CD8+ T cells could persist during the convalescent phase of the disease. In our study, the main changes in transcriptome are the decreased expression of MHC class II and type I interferon pathways in monocytes, but these were not associated with functional defects. In contrast, immune cells from convalescent individuals released more IFN-γ and IL-1Ra compared to healthy controls. The release of IFN-γ in individuals recovering from COVID-19 may reduce the susceptibility of host cells to secondary infections (49), while IL-1Ra might have an important role in the rebalancing of inflammatory responses. The discrepancy between transcriptomic and functional data may denote that we did not capture the entire functional spectrum of immune cells in our study, but may also underline the fact that cell function is likely regulated at multiple additional levels after gene transcription such as translation, processing, and post-transcriptional modifications (glycosylation, etc).

EWAS analysis reveals that, though not genome-wide significant, there is a preponderance of hypermethylated sites and down-regulated methylation in convalescent COVID-19 patients, explicitly originating from monocytes and CD4+ T cells. DNA methylation changes around DEGs in monocytes from scRNA-seq was not significantly different from the random set. This is likely due to the fact that the methylation was measured in the whole blood, which limits the power to detect the difference present in e.g. monocytes. In the future, further investigation focusing on monocytes may provide monocytes specific epigenetic change in convalescent individuals. Earlier studies have shown the incidence of hyper and demethylated CpG sites within COVID-19 patients: significant hypermethylation in regulatory regions of the genes related to the type I interferon response and first-line antiviral defense genes, like *ISG20* and *IFITM1*, are associated with disease severity in COVID-19 patients (14). Another study observed that systemic lupus erythematosus (SLE) patients are more likely to develop SARS-CoV-2 symptoms, not because of a weakened immune system, but because of overexpression of ACE2 in the lung, and hypomethylation of the *ACE2* gene, as well as a high level of demethylation of interferon genes (50). Retrotransposon upregulation, which is commonly experienced after coronavirus infection, is also reported to be possibly due to increased global DNA demethylation activity (51). These suggested that SARS-CoV-2 infection may have long-term effects, including irreversible genome modification among patients with prolonged recovery. Thus, genome-wide DNA methylation profiles of convalescent COVID-19 individuals are different from controls, which may be associated with clinical complication experienced by convalescent COVID-19 patients.

However, there are several limitations of this study to consider. Firstly, it is essential to note that the participants included in the study had no symptoms and they did not develop symptoms associated with the long term COVID-19 syndrome. Therefore, we could not associate the epigenetic and transcriptional signatures retained in convalescent patients with lingering symptoms, and these changes may not mirror functional effects. A recent study reported that 31 people with long COVID-19 symptoms exhibited less naïve T and B cells and higher plasma type I and type III interferons, even 8 months after the infection (52). Nevertheless, larger studies should explore whether these changes can be identified in larger populations and assess their clinical relevance. Our cohort mainly consisted of recovered COVID-19 patients from mild/moderate disease. The immunological differences we reported could be more apparent in the patients recovered from severe COVID-19. We have only one individual recovered from severe infection, however, we did not find differences compared with other participants in both transcriptome and methylome level (**Figures S7A–B**). Furthermore, this study was performed during the first wave of the pandemic, during which the original virus strain dominated the infections. Therefore, how the new variants affect the immune system after recovery remains elusive and must be investigated.

In summary, a comprehensive epigenetic, transcriptional and functional assessment shows that the immune responses of patients recovering from mild-to-moderate COVID-19 largely return to normal a few weeks to months after recovery. However, minor differences persist at the transcriptomic and epigenetic levels, especially in classical monocytes. Although MHC class II gene expressions and IFN response pathway were downregulated in monocytes of convalescent patients, those changes were not reflected in basal expressions of interferon-stimulated genes and cytokine production capacity of PBMCs. Therefore, our study shows that the immune system of patients who recover from mild/moderate COVID-19 largely return to normal, but future studies should investigate potential disturbances in individuals infected with different SARS-CoV-2 variants and patients suffering from long term COVID-19.

## Data Availability Statement

The datasets presented in this study have been deposited at the European Genome-phenome Archive (EGA), under accession number EGAS00001005529.

## Ethics Statement

The studies involving human participants were reviewed and approved by CMO Arnhem-Nijmegen (NL32 357.091.10). The patients/participants provided their written informed consent to participate in this study.

## Author Contributions

YL, MGN, and C-JX conceptualized and designed the study. ZL, GK, WL, BZ, OB, HP, YM, and CQ performed the data analysis supervised by YL, C-JX, and MGN. GK, OB, AVF, CM, and JD-A recruited the participants and collected the biological material. GK, OB, HC-T, ZL, AVF, CM, CS, JD-A, BC, HK, ES, DD, MJ, and MC performed or support the experiments. AvdV, FV, RC, BE-V, LJ, and MGN helped with participant recruitment and interpretation of the data. PO, LM, and HS provided the heat-inactivated SARS-CoV-2. YL, MGN, C-JX, ZL, GK, OB, and MG wrote the manuscript with input from all the authors. All authors contributed to the article and approved the submitted version.

## Funding

YL is supported by an ERC Starting Grant (948207) and a Radboud University Medical Centre Hypatia Grant (2018). MGN is supported by an ERC Advanced Grant (833247) and a Spinoza Grant of the Netherlands Organization for Scientific Research. C-JX is supported by the Helmholtz Initiative and Networking Fund (1800167). AVF is supported by the FSE and the Fundação para a Ciência e a Tecnologia (FCT, PhD grant PD/BD/135449/2017). LM, HS, and PO were supported by the Jürgen Manchot Foundation. This work was partly supported by the German Federal Ministry of Education and Research *“*NaFoUniMedCovid19*” *—COVIM [01KX2021]. ZL and WL were supported by a grant from the China Scholarship Council.

## Conflict of Interest

The authors declare that the research was conducted in the absence of any commercial or financial relationships that could be construed as a potential conflict of interest.

## Publisher’s Note

All claims expressed in this article are solely those of the authors and do not necessarily represent those of their affiliated organizations, or those of the publisher, the editors and the reviewers. Any product that may be evaluated in this article, or claim that may be made by its manufacturer, is not guaranteed or endorsed by the publisher.
