# Supplementary material for: Multi-Omics Integration Reveals Only Minor Long-Term Molecular and Functional Sequelae in Immune Cells of Individuals Recovered From COVID-19
Source: Front Immunol. 2022 Apr 6;13:838132. doi: 10.3389/fimmu.2022.838132 (PMC9022455; doi:10.3389/fimmu.2022.838132)
Supplement: Supplementary file 1 [file DataSheet_1.pdf]

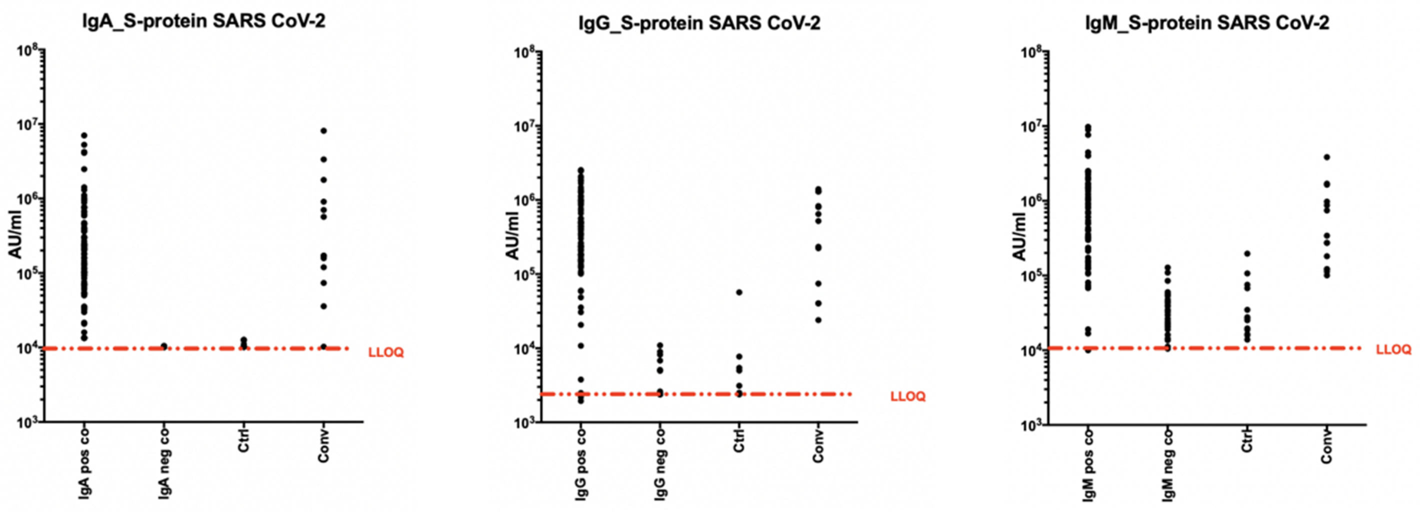

Fig. S1. IgA, IgG, and IgM antibodies against spike protein in the study participants.

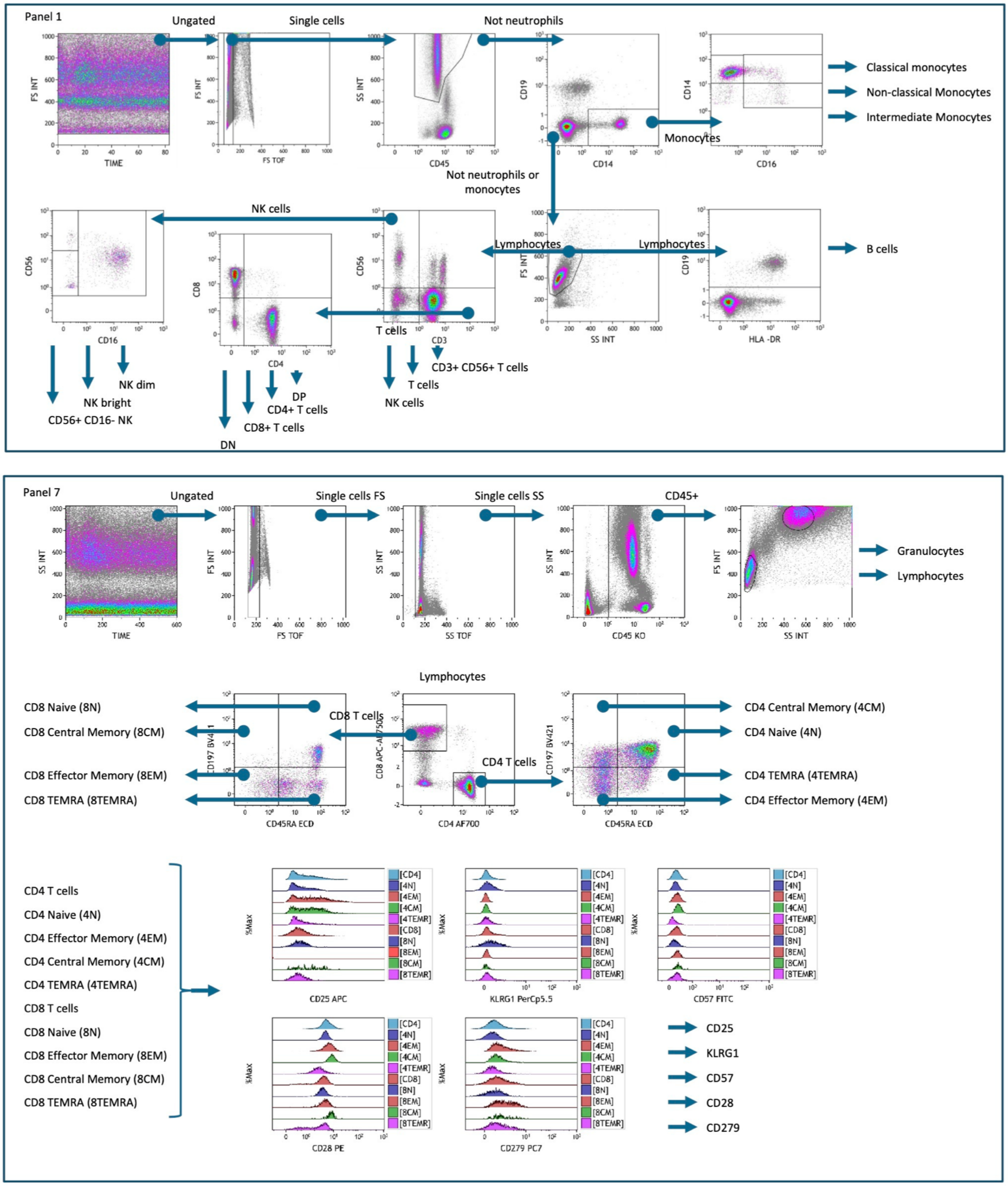

Fig. S2. Gating strategy used in flow cytometry analyses.

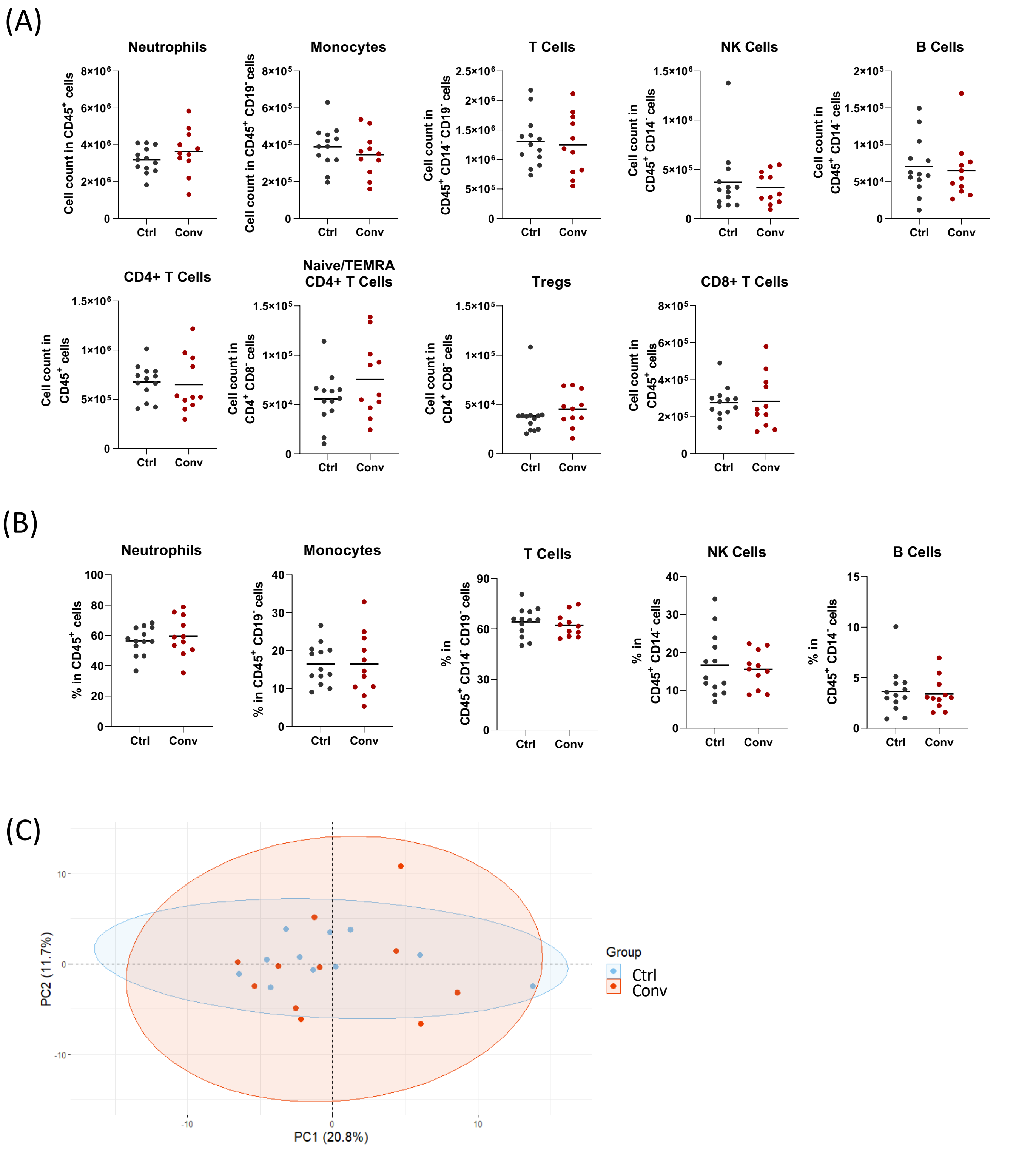

Fig. S3. Flow cytometry analysis of cell populations in the blood. (A) Dot plots showing the absolute cell counts of immune cell populations in the blood. (B) Dot plots showing the percentages of the main cell populations in the blood of healthy controls and convalescent COVID-19 patients. (C) Principal component analysis of the cell populations based on the absolute cell counts.

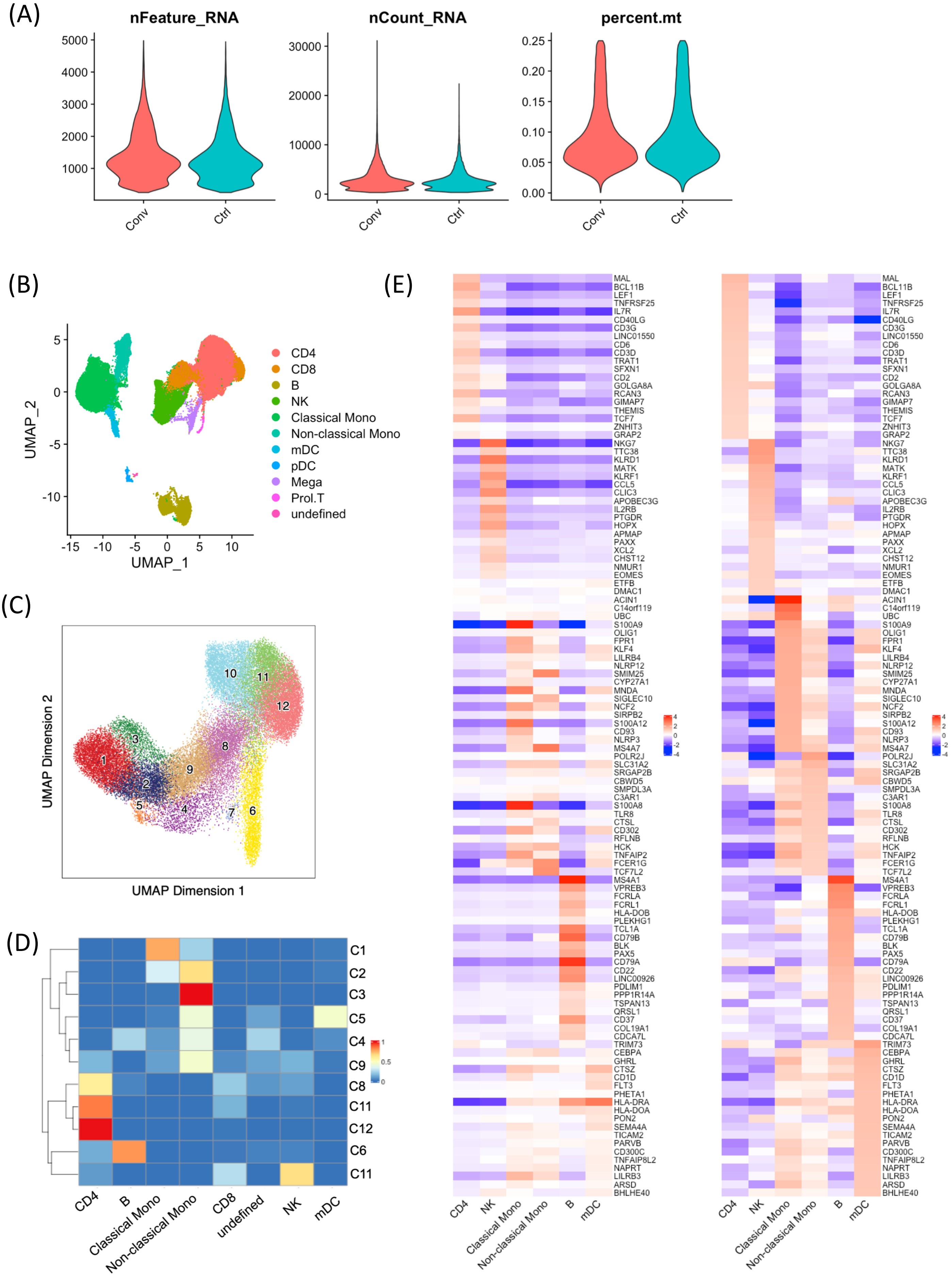

(A)
nFeature_RNA
nCount_RNA
percent.mt
Conv
Ctrl
(B)
UMAP_1
UMAP_2
CD4
CD8
B
NK
Classical Mono
Non-classical Mono
mDC
pDC
Mega
Prol.T
undefined
(C)
UMAP Dimension 1
UMAP Dimension 2
(D)
C1
C2
C3
C5
C4
C9
C8
C11
C12
C6
C11
CD4
B
Classical Mono
Non-classical Mono
CD8
undefined
NK
mDC
(E)
MAL
BCL11B
LEF1
TNFRSF25
IL7R
CD40LG
CD3G
LINC01550
CD6
CD3D
TRAT1
SFXN1
CD2
GOLGA8A
RCAN3
GIMAP7
THEMIS
TCF7
ZNHIT3
GRAP2
NKG7
TTC38
KLRD1
MATK
KLRF1
CCL5
CLIC3
APOBEC3G
IL2RB
PTGDR
HOPX
APMAP
PAXX
XCL2
CHST12
NMUR1
EOMES
ETFB
DMAC1
ACIN1
C14orf119
UBC
S100A9
OLIG1
FPR1
KLF4
LILRB4
NLRP12
SMIM25
CYP27A1
MNDA
SIGLEC10
NCF2
SIRPB2
S100A12
CD93
NLRP3
MS4A7
POLR2J
SLC31A2
SRGAP2B
CBWD5
SMPDL3A
C3AR1
S100A8
TLR8
CTSL
CD302
RFLNB
HCK
TNFAIP2
FCER1G
TCF7L2
MS4A1
VPREB3
FCRLA
FCRL1
HLA-DOB
PLEKHG1
TCL1A
CD79B
BLK
PAX5
CD79A
CD22
LINC00926
PDLIM1
PPP1R14A
TSPAN13
QRSL1
CD37
COL19A1
CDCA7L
TRIM73
CEBPA
GHRL
CTSZ
CD1D
FLT3
PHETA1
HLA-DRA
HLA-DOA
PON2
SEMA4A
TICAM2
PARVB
CD300C
TNFAIP8L2
NAPRT
LILRB3
ARSD
BHLHE40
CD4
NK
Classical Mono
Non-classical Mono
B
mDC

Fig. S4. Single-cell RNA-seq and ATAC-seq analysis of PBMCs in convalescent COVID-19 individuals. (A) Number of RNA features, counts, and mitochondria percentage after QC for the scRNA-seq dataset. (B) UMAP visualization of scRNA-seq profiling in both convalescent COVID-19 patients and controls. 11 cell clusters were annotated, each color indicated one cell type. (C) UMAP visualization of scATAC-seq profiling in both convalescence COVID-19 and controls. 12 cell clusters were identified. (D) Heatmap showing the integration results of scATAC-seq and scRNA-seq data. Color indicates the level of overlapping between two clusters from scATAC-seq and scRNA-seq. C1-C12 were corresponding to the cell clusters labelled in (C). (E) Heatmap showing the expression level of the top overlapping markers in scATAC-seq (left) and scRNA-seq (right).

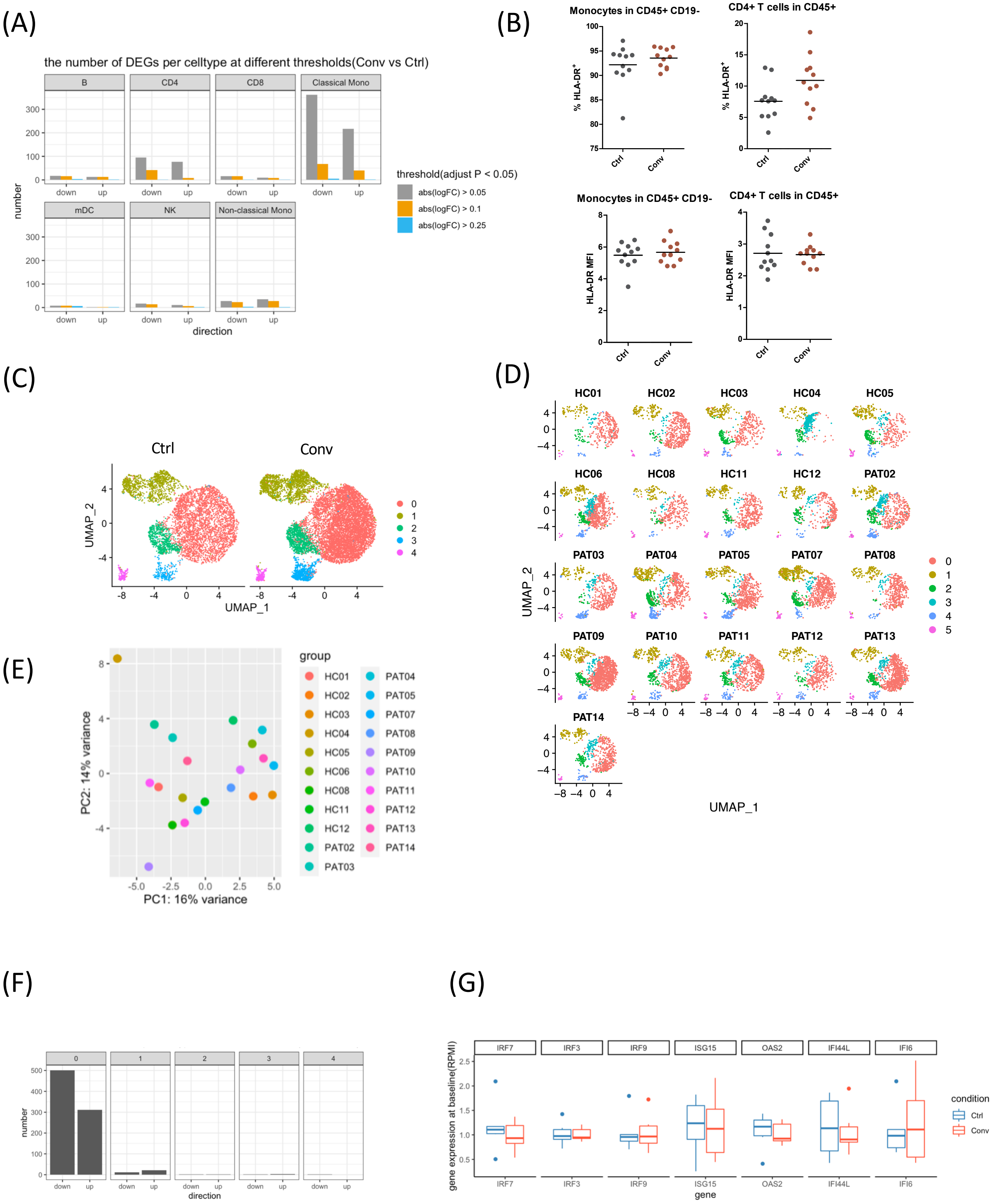
(A)
the number of DEGs per celltype at different thresholds(Conv vs Ctrl)
B
CD4
CD8
Classical Mono
mDC
NK
Non-classical Mono
number
direction
down
up
threshold(adjust P < 0.05)
abs(logFC) > 0.05
abs(logFC) > 0.1
abs(logFC) > 0.25
(B)
Monocytes in CD45+ CD19-
CD4+ T cells in CD45+
% HLA-DR+
HLA-DR MFI
Ctrl
Conv
(C)
Ctrl
Conv
UMAP_1
UMAP_2
(D)
HC01
HC02
HC03
HC04
HC05
HC06
HC08
HC11
HC12
PAT02
PAT03
PAT04
PAT05
PAT07
PAT08
PAT09
PAT10
PAT11
PAT12
PAT13
PAT14
UMAP_1
UMAP_2
(E)
group
PC1: 16% variance
PC2: 14% variance
(F)
number
direction
(G)
IRF7
IRF3
IRF9
ISG15
OAS2
IFI44L
IFI6
gene expression at baseline(RPMI)
gene
condition
Ctrl
Conv

Fig. S5. Transcriptional changes from single-cell RNA and ISG expression at baseline level. (A) The number of down- or up-regulated DEGs found in each cell type. The color for each bar depicts different thresholds of the log fold change. (B) Surface expression of HLA-DR in monocytes and CD4+ T cells, analyzed by flow cytometry. Both HLA-DR+ cells and the mean fluorescent intensity (MFI) of HLA-DR were shown. (C) UMAPs showing the sub-clusters in classical monocytes. (D) UMAPs showing the sub-clusters in classical monocytes split by samples. (E) PCA on classical monocytes specific-pseudo-bulk RNA result. (F) The number of DEGs between convalescence COVID-19 and controls were identified in each cluster. (G) Baseline ISG expressions level between controls and convalescent individuals.

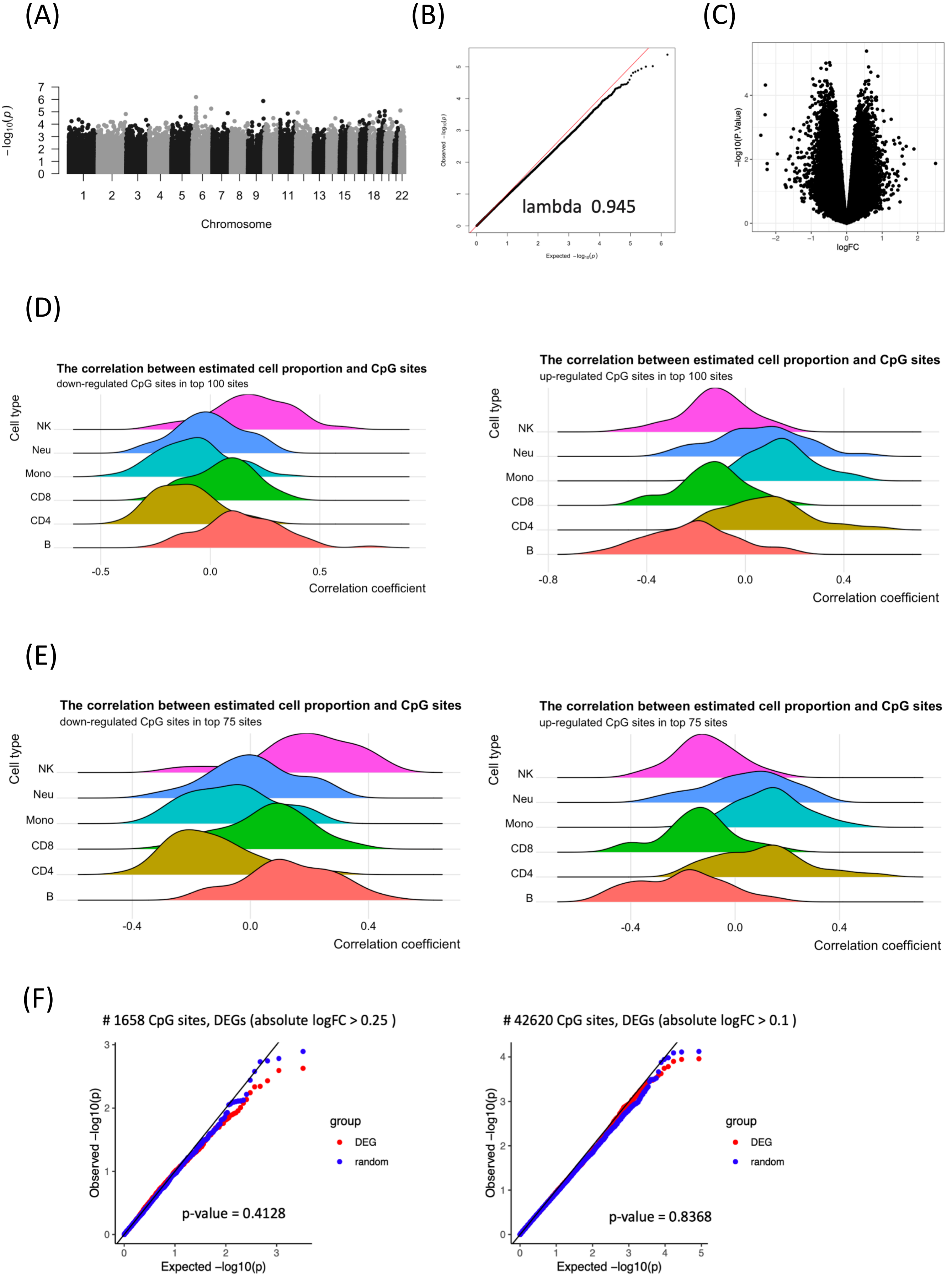

(A)
$-\log_{10}(p)$
7
6
5
4
3
2
1
0
1 2 3 4 5 6 7 8 9 11 13 15 18 22
Chromosome
(B)
Observed $-\log_{10}(p)$
Expected $-\log_{10}(p)$
lambda 0.945
(C)
−log10(P.Value)
logFC
(D)
The correlation between estimated cell proportion and CpG sites
down-regulated CpG sites in top 100 sites
Cell type
NK
Neu
Mono
CD8
CD4
B
-0.5
0.0
0.5
Correlation coefficient
The correlation between estimated cell proportion and CpG sites
up-regulated CpG sites in top 100 sites
Cell type
NK
Neu
Mono
CD8
CD4
B
-0.8
-0.4
0.0
0.4
Correlation coefficient
(E)
The correlation between estimated cell proportion and CpG sites
down-regulated CpG sites in top 75 sites
Cell type
NK
Neu
Mono
CD8
CD4
B
-0.4
0.0
0.4
Correlation coefficient
The correlation between estimated cell proportion and CpG sites
up-regulated CpG sites in top 75 sites
Cell type
NK
Neu
Mono
CD8
CD4
B
-0.4
0.0
0.4
Correlation coefficient
(F)
1658 CpG sites, DEGs (absolute logFC > 0.25)
Observed −log10(p)
Expected −log10(p)
group
DEG
random
p-value = 0.4128
42620 CpG sites, DEGs (absolute logFC > 0.1)
Observed −log10(p)
Expected −log10(p)
group
DEG
random
p-value = 0.8368

Fig. S6. Epigenome-wide association analysis of convalescent COVID-19 and healthy controls. (A) The Manhattan plot showing the epigenome-wide association analysis (EWAS) results comparing DNA methylation profiles between convalescent COVID-19 individuals and controls. The x-axis is the chromosomal position, and the y axis is the significance on a − log 10 scales. No significant CpG sites were identified with FDR < 0.05. (B) The quantile-quantile (Q-Q) plot showing the distribution of the -log10(*P value*) from the EWAS result and the expected -log10(*P value*). The lambda value is 0.945. (C) The volcano plot showing the log fold change and the -log10(*P value*) for each CpG site. (D) The density ridgeline plots showing the correction between the estimated cell proportions and the down-regulated(left) or up-regulated(right) CpG sites in the top 100 differentially methylated CpG sites. (E) The density ridgeline plots showing the correction between the estimated cell proportions and the down-regulated(left) or up-regulated(right) CpG sites in the top 75 differentially methylated CpG sites. (F) The quantile-quantile (Q-Q) plot showing the distribution of the -log10(P value) and the expected -log10(P value) for the CpG sites in the proximity (+/- 250kb) of the DEGs from monocytes and those random choose from our EWAS result. Red dot represented the CpG sites around DEGs, Blue dot represented the random chosen CpG sites. Two-sample Kolmogorov-Smirnov test was used to compare the distributions of the p values from two groups.

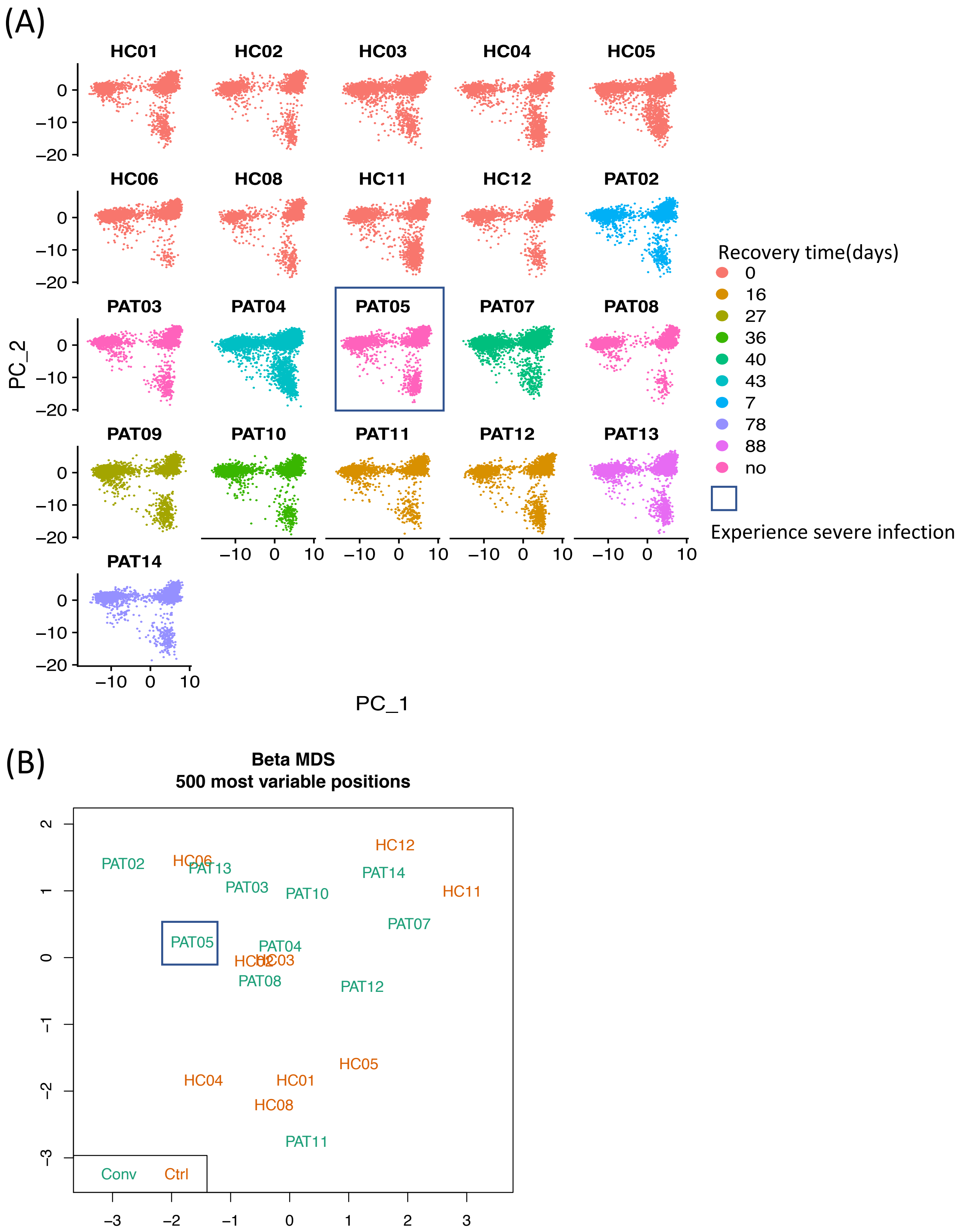
(A)
HC01
HC02
HC03
HC04
HC05
HC06
HC08
HC11
HC12
PAT02
PAT03
PAT04
PAT05
PAT07
PAT08
PAT09
PAT10
PAT11
PAT12
PAT13
PAT14
PC_2
PC_1
Recovery time(days)
0
16
27
36
40
43
7
78
88
no
Experience severe infection
(B)
Beta MDS
500 most variable positions
PAT02
HC06
PAT13
PAT03
PAT10
HC12
PAT14
HC11
PAT07
PAT05
PAT04
HC02
HC03
PAT08
PAT12
HC04
HC01
HC05
HC08
PAT11
Conv
Ctrl

Fig. S7. Dimensional reduction plot in single-cell RNA and methylation data. (A) PCA plot in each individual. Recovery time was defined as the disappearance of symptoms to sample collection. “0” represents the healthy control group. “no” in the legend indicates the missing value in our clinical information. (B) Multi-dimensional scaling (MDS) plots showing a 2-d projection of distances between participants from methylation data.

Table S1. Flow cytometry antibodies used for the analysis of immune cells

REAGENT or RESOURCE	SOURCE	IDENTIFIER
**Granulocyte panel**		
anti-CD16 FITC (clone 3G8)	Beckman Coulter	Cat#B49215; RRID: AB_2848116
anti-CD10 PE (HI10A)	BioLegend	Cat#312203; RRID: AB_314914
anti-CD11b PE-Dazzle (ICRF44)	BioLegend	Cat#301347; RRID: AB_2564080
anti-CD14 PE-Cy5.5 (clone M5E2)	BioLegend	Cat#301847; RRID: AB_2564058
anti-CD62L PE-Cy7 (clone DREG-56)	BioLegend	Cat#304821; RRID: AB_830800
anti-PD-L1 APC (clone MIH1)	ThermoFisher	Cat#17598342; RRID: AB_10597586
anti-CD66b AF700 (G10F5)	BioLegend	Cat#305113; RRID: AB_2566037
anti-CD15 Brilliant Violet 421 (clone W6D3)	BioLegend	Cat#323039; RRID: AB_2566519
anti-CD45 Krome Orange (clone J33)	Beckman Coulter	Cat#A96416; RRID: AB_2833027
**General panel**		
anti-CD16 FITC (clone 3G8)	Beckman Coulter	Cat#B49215; RRID: AB_2848116
anti-HLA-DR PE (clone immu-357)	Beckman Coulter	Cat#IM1639; RRID: AB_131284
anti-CD14 ECD (clone RM052)	Beckman Coulter	Cat#B92391; RRID: AB_130853
anti-CD4 PE-Cy5.5 (clone 13B8.2)	Beckman Coulter	Cat#B16491; RRID: Unknown
anti-CD25 PE-Cy7 (clone M-A251)	BD Biosciences	Cat#557741; RRID: AB_396847
anti-CD56 APC (clone N901)	Beckman Coulter	Cat#IM2474; RRID: AB_130791
anti-CD8 APC-AF700 (clone B9.11)	Beckman Coulter	Cat#B49181; RRID: AB_2750854
anti-CD19 APC-AF750 (clone J3-119)	Beckman Coulter	Cat#A94681; RRID: AB_2833030
anti-CD3 Pacific Blue (clone UCHT1)	Beckman Coulter	Cat#A93687; RRID: AB_2728095
anti-CD45 Krome Orange (clone J33)	Beckman Coulter	Cat#A96416; RRID: AB_2833027
**B cell panel**		
anti-IgD FITC (clone IADB6)	Southern Biotech	Cat#2032-02; RRID: AB_2687521
anti-IgM PE (clone SA-DA4)	Beckman Coulter	Cat#B30657; RRID: unknown
anti-CD3 ECD (clone UCHT1)	Beckman Coulter	Cat#A07748; RRID: unknown
anti-CD27 PE-Cy5.5 (clone 1A4CD27)	Beckman Coulter	Cat#B21444; RRID: unknown
anti-CD38 PE-Cy7 (clone LS198-4-3)	Beckman Coulter	Cat#B49198; RRID: unknown
anti-CD24 APC (clone ALB9)	Beckman Coulter	Cat#A87785; RRID: unknown
anti-CD5 APC-AF700 (clone BL1a)	Beckman Coulter	Cat#A78835; RRID: unknown
anti-CD19 APC-AF750 (clone J3-119)	Beckman Coulter	Cat#A94681; RRID: AB_2833030
anti-CD20 Pacific Blue (clone B9E9)	Beckman Coulter	Cat#B49208; RRID: unknown
anti-CD45 Krome Orange (clone J33)	Beckman Coulter	Cat#A96416; RRID: AB_2833027
**Senescence panel**		
anti-CD57 FITC (clone NC1)	Beckman Coulter	Cat#IM0466U; RRID: unknown
Anti-CD28 PE (clone CD28.1)	DAKO	Cat#R7164; RRID: AB_579570
Anti-CD45RA ECD (clone 2H4LDH11LDB9)	Beckman Coulter	Cat#B49193; RRID: unknown
Anti-KLRG1 PerCp-Cy5.5 (clone 2F1)	Biolegend	Cat#138418; RRID: AB_2563014
Anti-CD279 PE-Cy7 (clone EH12.2H7)	Biolegend	Cat#329918; RRID: AB_2159324
Anti-CD25 APC (clone 2A3)	BD Biosciences	Cat#340907; RRID: AB_2819021
Anti-CD4 AF700 (clone RPA-T4)	eBioscience	Cat#56-0049-42; RRID: AB_11219085
anti-CD8 APC-AF750 (clone B9.11)	Beckman Coulter	Cat#A94683; RRID: unknown
Anti-CD197 BV421 (clone G043H7)	Biolegend	Cat#353208; RRID: AB_11203894
anti-CD45 Krome Orange (clone J33)	Beckman Coulter	Cat#A96416; RRID: AB_2833027

Table S2. Human primers used for RT-qPCR

Gene	Primer	Sequence
HPRT1	Forward	CCTGGCGTCGTGATTAGTGAT
	Reverse	AGACGTTCAGTCCTGTCCATAA
IFI44L	Forward	AGGGAATCATTTGGCTCTGTAGA
	Reverse	AGCCGTCAGGGATGTACTATAAC
IFI6	Forward	GGTCTGCGATCCTGAATGGG
	Reverse	TCACTATCGAGATACTTGTGGGT
IRF3	Forward	AGAGGCTCGTGATGGTCAAG
	Reverse	AGGTCCACAGTATTCTCCAGG
IRF7	Forward	GCTGGACGTGACCATCATGTA
	Reverse	GGGCCGTATAGGAACGTGC
IRF9	Forward	GATACAGCTAAGACCATGTTCCG
	Reverse	TGATACACCTTGTAGGGCTCA
ISG15	Forward	CGCAGATCACCCAGAAGATCG
	Reverse	TTCGTCGCATTTGTCCACCA
OAS2	Forward	CTCAGAAGCTGGGTTGGTTTAT
	Reverse	ACCATCTCGTCGATCAGTGTC

Table S3. Conditions of the RT-qPCR reaction

Step	Temperature	Duration	Cycle #
Initial Denaturation	95°C	10 minutes	1
Denaturation	95°C	15 seconds	40
Annealing/Extension	60°C	60 seconds	
Hold	4°C	∞	

Table S4. Significant differential methylation regions between convalescent individuals and controls.

chr	start	end	p	fdr	sidak	nprobe
4	184908582	184909019	3,44855E-12	1,03457E-10	6,26732E-09	7
6	32063726	32064498	1,83542E-10	2,75312E-09	1,88817E-07	19
7	51539131	51539584	2,4409E-09	2,4409E-08	4,27933E-06	5
6	26195697	26195996	8,23619E-09	6,17714E-08	2,18763E-05	4
10	18689430	18689949	2,70612E-08	1,6208E-07	4,1409E-05	8
20	26190328	26190355	3,24159E-08	1,6208E-07	0,000953041	3
17	1808573	1808744	4,10798E-08	1,76056E-07	0,000190772	3
6	31275551	31275808	8,22663E-08	3,08498E-07	0,000254189	8
8	61880392	61880471	1,05629E-07	3,52096E-07	0,001061324	3
1	77230619	77230960	1,46924E-07	4,15219E-07	0,000342128	4
1	228291486	228291706	1,52247E-07	4,15219E-07	0,000549453	4
16	6534204	6534431	2,02085E-07	5,05212E-07	0,000706771	3
11	133928292	133928347	5,2837E-07	1,21932E-06	0,007600526	3
2	128453260	128453485	5,8092E-07	1,24483E-06	0,002048389	4
17	36997449	36997741	1,05346E-06	2,10691E-06	0,00286112	7
1	2501853	2501938	1,38444E-06	2,59582E-06	0,012852065	2
6	34482411	34482680	1,49014E-06	2,62966E-06	0,004389796	6
6	116381904	116382180	1,79163E-06	2,98606E-06	0,005142156	5
18	3624189	3624430	2,17096E-06	3,42783E-06	0,007128637	2
1	203044930	203045230	2,94096E-06	4,41144E-06	0,007755376	3
14	106322760	106322987	3,56075E-06	5,08678E-06	0,01238048	3
3	9289747	9289894	3,90732E-06	5,32816E-06	0,020888631	3
11	3190573	3190639	4,98267E-06	6,49913E-06	0,058195365	2
12	67197851	67197985	5,66787E-06	7,08484E-06	0,033034402	3
15	66947564	66947618	3,94539E-05	4,73447E-05	0,440251472	3
12	132293388	132293657	4,15007E-05	4,78854E-05	0,11531907	3
16	67997858	67997922	0,000584899	0,000649888	0,999297071	2
10	375391	375392	0,002219619	0,002378163	1	1
5	38445612	38445835	0,002607366	0,002697275	0,999908389	4
19	55973102	55973112	0,003353333	0,003353333	1	2
